# Quantitative dual-energy CT as a nondestructive tool to identify indicators for fossilized bone in vertebrate paleontology

**DOI:** 10.1038/s41598-022-20707-5

**Published:** 2022-09-30

**Authors:** Charlie A. Hamm, Oliver Hampe, Jürgen Mews, Christina Günter, Ralf Milke, Florian Witzmann, Lynn J. Savic, Lutz Hecht, Sabine Meister, Bernd Hamm, Patrick Asbach, Torsten Diekhoff

**Affiliations:** 1 Department of Radiology, Charité-Universitätsmedizin Berlin, Corporate Member of Freie Universität Berlin, Humboldt-Universität zu Berlin, and Berlin Institute of Health, 10117 Berlin, Germany; 2 Institute of Diagnostic Radiology and Neuroradiology, Greifswald University Hospital, Ferdinand-Sauerbruch-Straße, 17475 Greifswald, Germany; 3 Museum für Naturkunde, Leibniz-Institut für Evolutions- und Biodiversitätsforschung, Invalidenstraße 43, 10115 Berlin, Germany; 4 Canon Medical Systems Europe BV, Global RDC, Zilverstraat 1, 2718RP Zoetermeer, The Netherlands; 5 Institute for Geosciences, University of Potsdam, 14469 Potsdam, Germany; 6 Institut für Geologische Wissenschaften, Freie Universität Berlin, Malteserstraße 74-100, 12249 Berlin, Germany; 7 Berlin Institute of Health at Charité—Universitätsmedizin Berlin, Berlin, Germany

**Keywords:** X-ray tomography, Structure determination, Palaeontology, Palaeontology

## Abstract

Dual-energy computed tomography (DECT) is an imaging technique that combines nondestructive morphological cross-sectional imaging of objects and the quantification of their chemical composition. However, its potential to assist investigations in paleontology has not yet been explored. This study investigates quantitative DECT for the nondestructive density- and element-based material decomposition of fossilized bones. Specifically, DECT was developed and validated for imaging-based calcium and fluorine quantification in bones of five fossil vertebrates from different geological time periods and of one extant vertebrate. The analysis shows that DECT material maps can differentiate bone from surrounding sediment and reveals fluorine as an imaging marker for fossilized bone and a reliable indicator of the age of terrestrial fossils. Moreover, the jaw bone mass of *Tyrannosaurus rex* showed areas of particularly high fluorine concentrations on DECT, while conventional CT imaging features supported the diagnosis of chronic osteomyelitis. These findings highlight the relevance of radiological imaging techniques in the natural sciences by introducing quantitative DECT imaging as a nondestructive approach for material decomposition in fossilized objects, thereby potentially adding to the toolbox of paleontological studies.

## Introduction

Multiple studies have investigated fragmented bone specimens reporting varying elementary compositions of bony tissue of fossil objects^1–3^. Those variations can be explained, inter alia, by the concept of diagenesis as hydroxyapatite in bones and teeth is replaced by thermodynamically more stable fluorapatite during the process of fossilization^4–6^. This process relies on the absorption of fluoride ions by the bone mineral when exposed to groundwater or soil^7,8^. The uptake of fluorine into bone apatite within the geological environment is interpreted as an exchange mechanism by coupled dissolution and precipitation, thus preserving the orginal shape of the object^9,10^. Uptake of fluorine by diffusion into solid media is excluded at the low temperatures of diagenesis based on grain boundary diffusion data^11^ and specifically also crystal diffusion data for apatite^12^. Therefore, fluorine must have been delivered by intergranular fluid within the solidifying sediment and was incorporated into bone by a dissolution-crystallisation process. Given the higher chemical stability of fluorapatite as compared to hydroxyapatite, hydroxyl ions in the bone mineral are replaced by fluoride over time^7^. However, at present, there is no physical method that enables noninvasive chemical characterization of fossil objects. In this regard, CT imaging has become valuable in the field of vertebrate paleontology given its ability to measure even very dense objects in a nondestructive manner^13,14^. Specifically, three-dimensional reconstructions and illustrations of the anatomy of fossilized bones generated by clinical CT scanners and micro-CT have contributed significantly to this field. In recent years, several CT analyses of fossilized specimens have been the basis for addressing higher-level scientific issues, such as the evolutionary development, physiology, and precise and detailed characterization of diseases in extinct animals^13,15–19^.

During the past few decades, continuous technical advancement has led to a substantially improved spatial resolution of conventional CT imaging, allowing for surface rendering in addition to obtaining information from the inside of investigated objects^13,20^. While conventional CT measures the X-ray attenuation in Hounsfield units (HU), spectral or dual-energy (DE)CT provides additional information^21,22^. As the effective atomic number (Z_eff_) influences two important absorption effects, namely, the photo-electric effect and Compton scattering, material absorption is dependent on the energy of the X-ray photon^23,24^. While the photo-electic effect is dominant for the attenuation of low-energy X-rays, the Compton effect increases with increasing energy^25,26^. Both effects depend to a different degree on the atomic number (Z), which in turn enables an estimation of the Z_eff_ using both measurements. Therefore, the application of two or more X-ray spectra in dual- or multi-energy CT allows for the application of a two-material algorithm for the differentiation of materials and objects larger than the spatial resolution (e.g., in gout imaging^27^) or a three-material algorithm to virtually decompose a single voxel into three materials using their respective spectral characteristics^28^. This technique is comparable to other spectroscopy techniques.

In addition to human medicine, DECT has found several applications in the natural sciences given these properties, such as hydrology, geology, and mineralogy^29–31^. Moreover, DECT has proven useful in assisting morphological studies of human history, e.g. by revealing new aspects of the materials used for mummification in Egypt in 800 BCE^32^. However, the potential applicability of DECT imaging in the field of vertebrate paleontology has not yet been fully explored^33^ but may provide substantial benefits due to the nondestructive detection, illustration, and quantitative characterization of fossilized bone and its chemical composition. In particular, specific chemical diagenetic processes could serve as targets for the development of age- and bone-specific indicators in paleontology using DECT imaging.

Therefore, the purpose of this study was to develop and validate density- and element-based material decomposition using nondestructive DECT to identify quantitative imaging indicators for fossilized bone and osseous disease characteristics in paleopathology.

## Methods

### Experimental design

**Figure 1:**
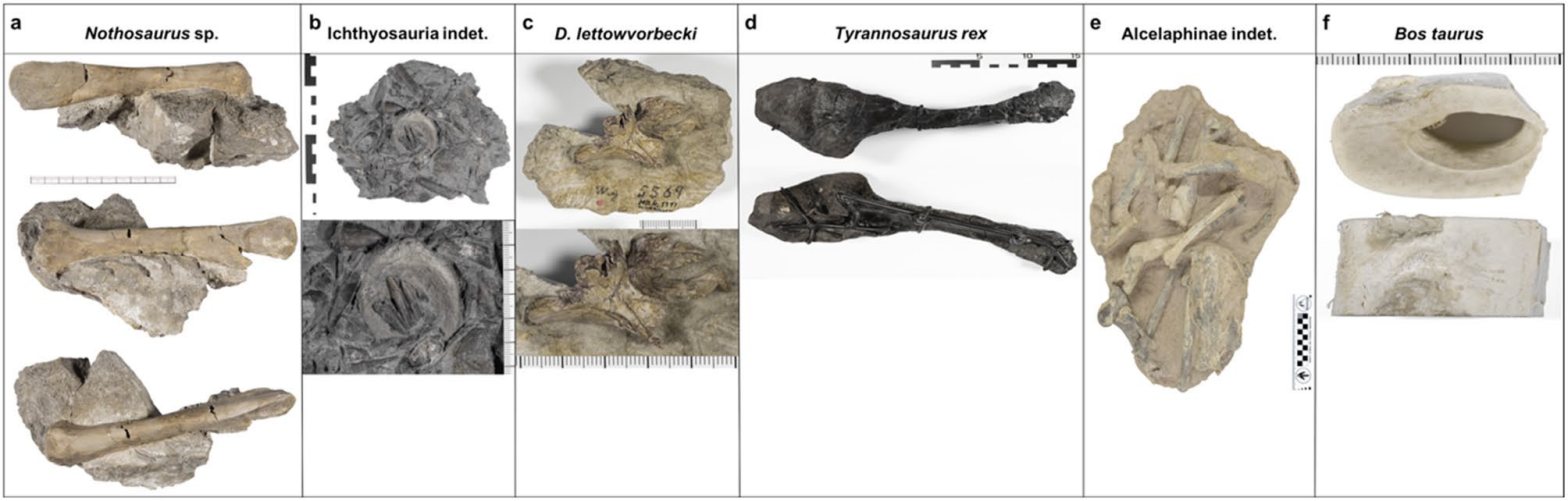
Photographs of investigated objects. In the horizontal, the objects are divided into marine and terrestrial animals and listed in order of their geological time periods. Photographs of the investigated marine (**a**,**b**) and terrestrial (**c**–**f**) objects. Photographs of the calibration objects, the haemal arch of the *Tyrannosaurus rex* MB.R. 5742.1 and leg bone of *Bos taurus* MB.Ma. 52910 (**d**,**f**), were taken after bone fragment removal. The test objects include *Nothosaurus* sp. MB.R. 2830 and Ichthyosauria indet. MB.R. 3538.3 (**a**,**b**) and *Dysalotosaurus lettowvorbecki* MB.R. 1317 and Alcelaphinae indet MB.Ma. 51906 (**c**,**e**).

**Figure 2:**
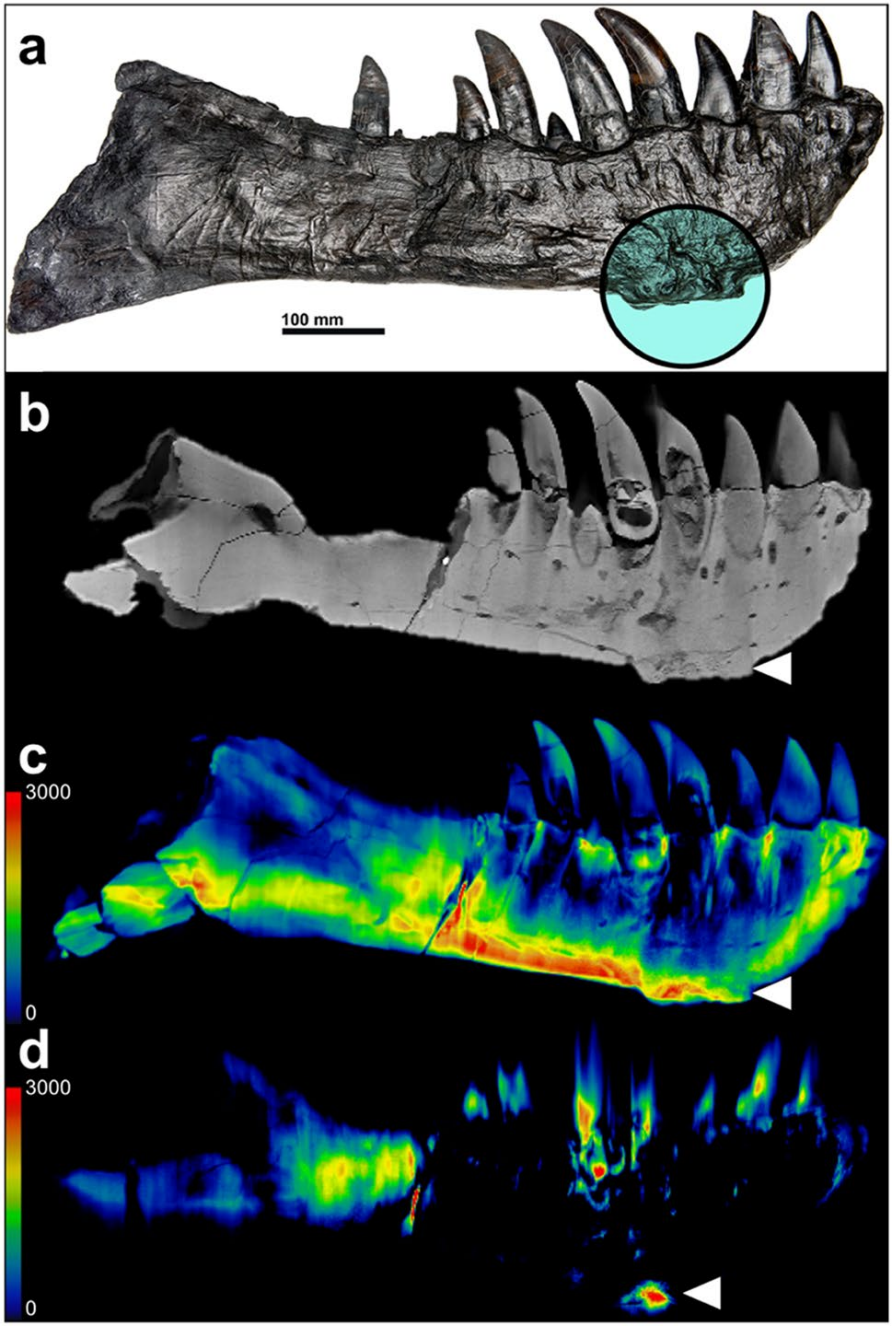
Diseased left dentary of the *Tyrannosaurus rex* MB.R. 91216. Photograph (**a**), conventional CT-reconstruction (**b**) and DECT-based calcium (**c**) and fluorine maps (**d**) of the left dentary in sagittal orientation. The circle in (**a**) and the arrowheads in (**b**–**d**) indicate the focal exophytic mass on the ventral bone surface at the level of the 3rd to 5th tooth-root. The DECT-material maps demonstrate a diffuse and heterogenous calcium concentration (**c**) but a focal fluorine accumulation in the central part of the exophytic mass (**d**). Red indicates high concentrations of the investigated element measured in Hounsfield units (color bar in the left lower corner).

This study was performed on fossilized bones from five different vertebrate taxa and one extant vertebrate. Seven specimens were selected and scanned with DECT: two calibration objects (an unfossilized *Bos taurus* leg bone and a *Tyrannosaurus rex* (*T. rex*) haemal arch) and five additional test objects (fossilized bones of *Nothosaurus* sp., Ichthyosauria indet., *Dysalotosaurus lettowvorbecki*, Alcelaphinae indet., and the diseased left dentary of a *T. rex;* Figs. 1 and 2). First, aiming to identify indicators for fossilized bone which could serve as an target for DECT material quantification, multimodal bone analysis was performed on selected “fresh” and fossilized objects. The elementary compositions of the two calibration objects were analyzed using multimodal bone analysis comprising X-ray diffractometry, scanning electron microscopy coupled with energy disperse X-ray spectrometry, optical emission spectrometry and elemental analysis. Secondly, those elements detected in the multimodal bone analysis were selected for DECT postprocessing with a quantitative three-material decomposition algorithm. Finally, DECT imaging of the five fossil test objects was performed and the previously developed algorithm was applied to assess their elementary composition and the pathology of the left dentary bone of the *T. rex*. A detailed description of the multimodal bone analysis is provided in the supplementary information S1.

### Investigated objects

*Calibration objects*. Specimens used for DECT calibration were the haemal arch of an anterior caudal vertebra (ca. 3.-5. Ca) of the *T. rex* “Tristan Otto” MB.R. 5742.1 and the leg bone of a *Bos taurus* MB.Ma. 52910 (Fig. 1). The *T. rex* is from the Maastrichtian (Late Cretaceous) and approximately 68 million years old and was unearthed in SE Montana (Carter County), USA. This specimen is in excellent condition, with 170 fossilized bones preserved and a nearly complete three-dimensionally preserved skull^13^. The haemal arch examined here is entirely black, as is the rest of the remaining skeleton, and has a length of 31.5 cm, a maximum width of 10.4 cm and a maximum depth of 6.6 cm. A steel frame is attached to the dorsal side of the object for stabilization. For bone matrix analysis, a bone fragment measuring 5.5 × 3 × 2 cm was detached from the proximal portion of the bone (Fig. S1). The leg bone MB.Ma. 52910 (*Bos taurus*; extant) from Bückeburg, Lower Saxony, Germany, measured 2.5 × 6.1 × 3.2 cm. For bone matrix analysis, the bone was kept frozen until defragmentation and subsequent bone analysis. Both objects were scanned before bone defragmentation, and the haemal arch was additionally scanned after bone fragment removal to demonstrate the exact location where the bone fragment was removed.

#### Sediment-embedded test objects

The developed DECT algorithm was tested for its potential to detect specific element concentrations through imaging of the sediment-embedded fossilized bones of two marine reptiles (*Nothosaurus* sp. and Ichthyosauria indet.) and two terrestrial animals (*Dysalotosaurus lettowvorbecki* and Alcelaphinae indet.) (Fig. 1): a femur of *Nothosaurus* sp. MB.R. 2830 (Bad Sulza Formation, upper Muschelkalk, Illyrian, late Anisian, Middle Triassic of Bad Sulza, Thuringia, Germany; approximately 242 Ma), a vertebral centrum of Ichthyosauria indet. MB.R. 3538.3 (*aalensis* Zone, late Toarcian (Lias ζ), Early Jurassic of Mistelgau clay pit, near Bayreuth/Upper Franconia, Germany; approximately 175 Ma), a parietal bone of *Dysalotosaurus lettowvorbecki* MB.R. 1317 (Middle Dinosaur Member, late Kimmeridgian, Late Jurassic of Kindope stream bed, Tendaguru, Lindi Region, Tanzania; approximately 153 Ma), and limb bones of Alcelaphinae indet. MB.Ma. 51906 (Bed II, Pleistocene of Olduvai Gorge, Arusha Region, N Tanzania; approximately 1.7–1.3 Ma). All specimens are stored in the Museum für Naturkunde Berlin, Germany.

#### *Diseased Left Dentary of the T. rex “Tristan Otto” *(digital dataset MB.R. 91216)

The left dentary shows a diffuse enlargement with thickening of nearly the entire left dentary and a focal exophytic mass on the ventral surface of the dentary (Fig. 2). The diffuse thickening extends from the tip of the dentary ventrally into the non-tooth bearing area with a total anterior–posterior expansion of approx. 43 cm. The ventral surface of the left dentary shows a focal exophytic mass measuring 113 mm, a prominence of 14 mm, a mediolateral width of 47 mm with a central depression of 8 mm and a rough and irregular surface.

### DECT calibration

Based on the findings from the multimodal bone analysis, a comprehensive panel of calibration elements was selected for DECT calibration. To enable the calibration of the DECT and to build a validated quantitative image analysis algorithm, selected calibration elements were chosen with the aim of demonstrating a high density and concentration of the investigated element. Specifically, calibration elements in the solid aggregate state were selected if they constituted more than 1% of the total bone mass of the *T. rex* haemal arch.

#### DECT calibration: imaging protocol

DECT was performed on a medical 320-row CT scanner (Aquilion ONE—Vision Edition, Canon Medical Systems Corp.) with an X-ray absorption measurement range from − 32,768 to + 32,767 HU. The dual-energy acquisition followed the rotate/rotate principle, which uses a sequential acquisition at both energies by applying a full gantry rotation, each, without table movement^34^. The volume acquisition collimation was 320 × 0.5 mm. The tube currents were set to 100 mA (135 kVp) and 570 mA (80 kVp) while applying a gantry rotation time of 1.0 s.

#### DECT calibration: image analysis

By applying quantitative measurements on the generic high- and low-kVp images, the dual-energy properties (densities in high and low kVp datasets and the corresponding material-specific gradient) of each investigated calibration material were used to adjust the parameters of a three-material decomposition algorithm. Finally, algorithms were developed to nondestructively detect and quantify selective elements on imaging.

### DECT imaging of calibration and test objects

The scans were performed at the same clinical scanner using sequential dual-energy helical acquisition that allows for scanning of specimens exceeding the 16 cm z-axis coverage of the detector. Here, two separate and sequential spiral scans were used, one high-energy and one low-energy acquisition, to image the specimen. The protocol was performed with a slow pitch of 0.41, a collimation of 80 × 0.5 mm and tube currents of 200 mAs (135 kVp) and 700 mAs (80 kVp) for large objects (e.g. left dentary of *T. rex*) and 150 mAs (135 kVp) and 400 mAs (80 kVp) for smaller objects (e.g. *Dysalotosaurus lettowvorbecki)*. The gantry rotation time was 0.75 s.

#### Image reconstruction

The dual-energy datasets were reconstructed with a thickness of 0.5 mm and a reconstruction interval of 0.3 mm using a medium soft-tissue kernel without beam hardening compensation and a sharp bone kernel. Multiplanar reconstructions were reconstructed with 0.5 mm isometric voxel using built-in postprocessing software on the CT console. The datasets were then transferred to a dedicated research PC for further postprocessing using dual-energy image view V6.00 SP 0500E (Canon Medical Systems Corp.).

A three-material decomposition algorithm was applied for the nondestructive detection and quantification of selected elements during imaging and the reconstruction of DECT material maps.

#### Quantitative image analysis

Quantitative image analysis was carried out using the imaging datasets of the DECT scans of each investigated object. The HU values of the 135 kVp conventional CT datasets and the HU values of the DECT material-maps were measured by assigning ten structure-specific regions of interest (ROIs) in (a) the bony tissue and (b) associated sediment of the two calibration objects and four sediment-embedded test objects and (c) the healthy and pathologic bony tissue of the left dentary of the *T. rex,* respectively. The ROIs were placed in representative areas of the object with few artefacts from beam hardening or photon starvation based on a consensus of two radiologists. ROIs free of obvious artefacts were included in the analysis, and the HU values were recorded for further statistical analysis. Calcium and fluorine contents were quantified using DECT three-material decomposition and illustrated using a color-map overlay.

### Statistical analysis

Descriptive results are reported as mean ± standard deviation (SD), median and range. Comparative analyses were performed using parametric one-way ANOVA with Bonferroni and Tukey post hoc testing to compare detected mineral amounts in the bone vs. sediment, healthy vs. diseased bone tissue and among tested specimens, respectively. Two-sided p values < 0.05 were considered statistically significant. Statistical analyses were performed using SPSS (v24.0, IBM Corp.) and Prism (v7.0, Graphpad).

## Results

### Fluorine as an indicator of fossilized bone in multimodal bone analysis

The quantitative multimodal bone analyses of both calibration objects revealed substantial differences in composition (Table 1; Supplementary Information S1). In the *T. rex* specimen, fluorapatite was the predominate mineral with an apatite phase of 96.40 wt. %, a total fluorine content of 4.16 wt. %, and iron content of 0.82 wt. %. In contrast, the observed mineral ratios in the *Bos taurus* (apatite phase of 75.57 wt. %, fluorine of < 0.05 wt. %, and iron of 0.01 wt. %) reflect hydroxyapatite as the predominant mineral, which is characteristic of “fresh” tissue. In summary, the results of the multimodal bone analysis revealed fluorapatite as a potential indicator for permineralized bone tissue, whereas hydroxyapatite is a potential indicator of rather “fresh” bone tissue.

**Table 1:** Relevant elements detected in the *Tyrannosaurus rex* haemal arch and *Bos taurus* leg bone and their DECT characteristics.

Element	*T. rex* in wt. % (Ca_5_ (PO_4_,CO_3_)_3_F)	*Bos taurus* in wt. % (Ca_5_(PO_4_)_3_OH)	DECT calibration materials			DECT values		
			Name	Formula	*Z*_eff_*	80 kVp	135 kVp	Quotient
Apatite phase (incl. F)	96.40 (3.48)	75.57 (n.d.)	Calcite crystal	CaCO_3_	15.88	4248	3000	0.707
Remaining F	0.68	< 0.05	Fluorite crystal	CaF_2_	16.42	5432	3753	0.691
CO_2_	1.43	4.97	n.a	n.a	n.a	n.a	n.a	n.a
H-	0.45	1.32	n.a	n.a	n.a	n.a	n.a	n.a
TOC	1.91	10.91	Graphite cylinder	C	6	325	381	0.85
SO_3_	1.14	0.18	Sulfur crystal	S	16	3196	2153	0.674
Fe_2_O_3_	0.82	0.01	Iron rod	Fe	26	24,422	18,311	0.75
Na_2_O	0.75	0.66	Sodium chloride	NaCl	13.97	1569	939	0.598
Al_2_O_3_	0.41	0.20	n.a	n.a	n.a	n.a	n.a	n.a
MnO	0.29	n.d	n.a	n.a	n.a	n.a	n.a	n.a
MgO	0.16	0.64	n.a	n.a	n.a	n.a	n.a	n.a
N	0.11	3.98	n.a	n.a	n.a	n.a	n.a	n.a
SiO_2_	0.10	0.12	n.a	n.a	n.a	n.a	n.a	n.a
Cu	< 0.01	n.d	n.a	n.a	n.a	n.a	n.a	n.a

The effective atomic number (Z_eff_) was calculated by a professional chemist.

*n.d.* not detectable, *n.a.* not analyzed.

### Selection of DECT calibration elements and development of the three-material decomposition algorithm for the detection of calcium and fluorine

The multimodal bone analysis revealed calcium, fluorine, carbon, sulfur, iron, and sodium as the most common elements in the apatite phase. Calibration materials were chosen accordingly as detailed in Table 1.

Based on these results, a three-material decomposition algorithm was chosen for quantitative DECT analysis of calcium and fluorine in bone. The following material formulas and gradients were applied: a build-in calcium-specific algorithm from clinical practice and a custom-made algorithm for fluorine with the three elements: calcium (4248 HU and 3000 HU at 80 kVp and 135 kVp, respectively), iron (10,000 HU and 7365 HU at 80 kVp and 135 kVp, respectively) and a gradient of 0.69 for fluorine (Fig. S2).

### Quantitative DECT image analysis revealed a significantly high fluorine content in permineralized bone tissue

**Figure 3:**
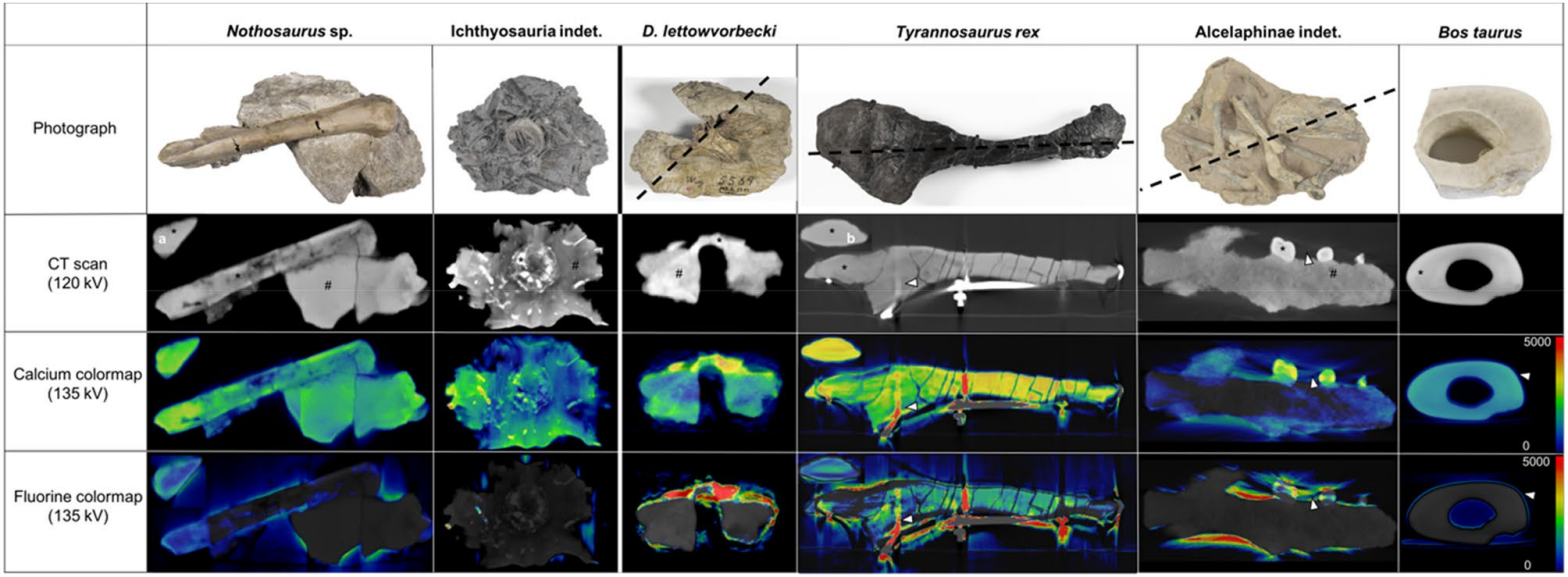
Photographs and CT images and DECT material maps of bone samples from the calibration and the sediment-embedded test objects. In the horizontal, the objects are divided into marine and terrestrial animals and listed in order of their geological time periods. The photographs and the black dotted lines in the first row indicate the cross-sectional plane of the CT and DECT images of the respective object. The photograph of the *Bos taurus* leg bone was taken after bone fragment removal. In the second row, bone tissue is indicated with a black asterisk (*), and sediment is indicated with a black hashtag (#). DECT material maps show detected calcium (third row) and fluorine (fourth row) in the investigated specimens. Red indicates high concentrations of the investigated element measured in Hounsfield units (color bar in the right lower corner). As the *Tyrannosaurus rex (T. rex)* and *Nothosaurus* sp. specimens were relatively large compared to the other specimens, additional axial planes are illustrated to show the calcium and fluorine distribution within the bone (**a**,**b**, respectively). The demonstrated axial plane of the *T. rex* specimen is at the level of the bone fragment, which was removed for invasive bone analysis, allowing for image-mass spectrometry correlation. White arrowheads indicate observed artefacts, partly caused by the attached metal frame (haemal arch of *T. rex*).

The calibration objects (haemal arch of the *T. rex* and leg bone of *Bos taurus*) demonstrated a homogenous bone texture without evidence of pathologic bone alterations (Fig. 3).

**Figure 4:**
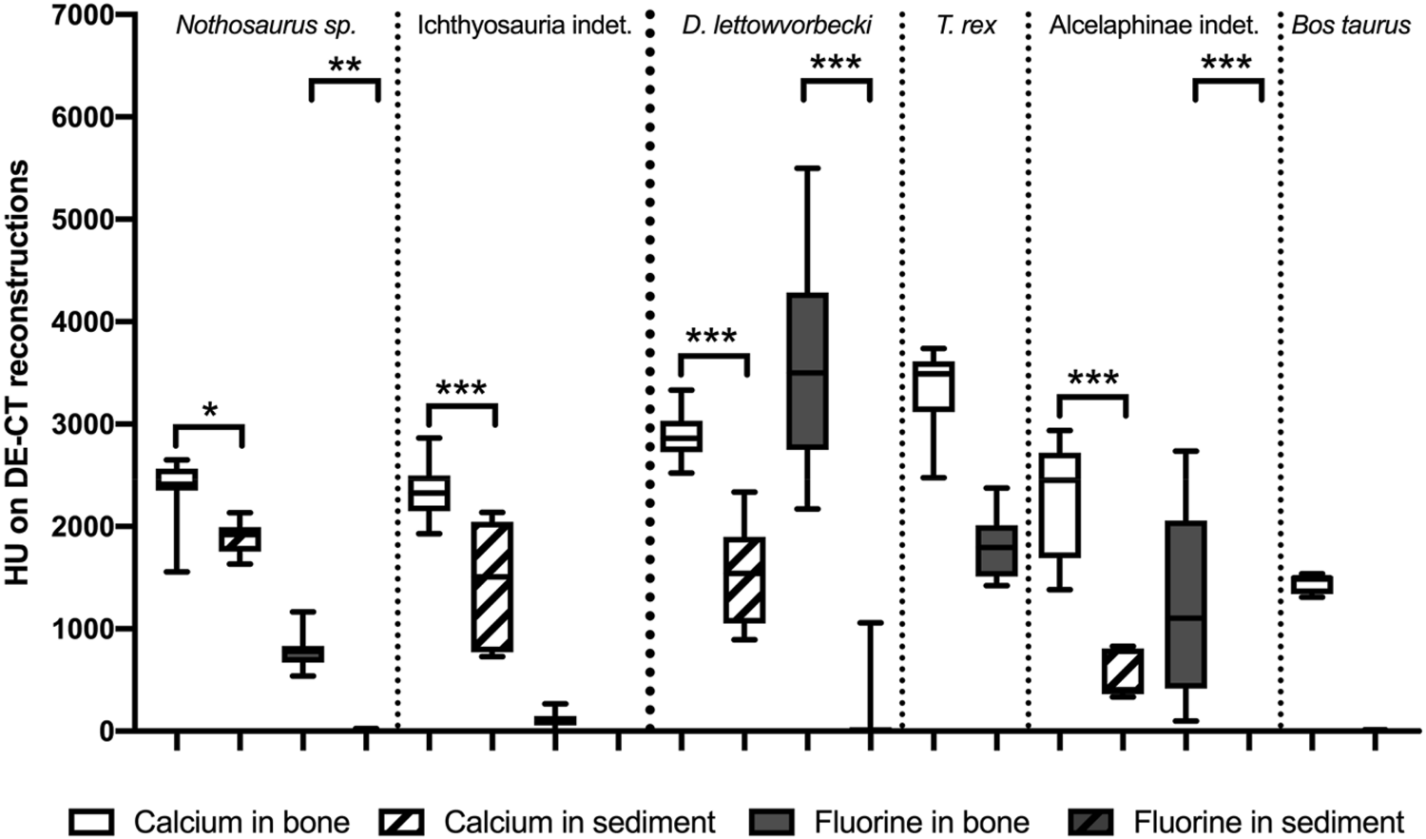
Nondestructive calcium and fluorine quantification in the bone and sediment using DECT. In the horizontal, the objects are divided into marine and terrestrial animals and listed in order of their geological time periods. Quantitative assessment of detected calcium and fluorine in DECT reconstructions in bone and sediment of the investigated specimen. DECT imaging revealed a difference in fluorine and calcium content in the *Tyrannosaurus rex* and *Bos taurus* specimens (p < 0.001). While the low fluorine content in the bone and sediment of the ichthyosaur was the exception, differences in calcium and fluorine in bone and sediment were significant in each sediment-embedded specimen. Significant differences between the bone and sediment are indicated by an asterisk (*p = 0.017; **p = 0.003; ***p < 0.001).

Artefacts in the dorsal parts of the haemal arch were caused by a metal frame and beam-hardening artefacts were observed near the surface. A mean HU of 3040 ± 542 (median 3217 HU; range, 1940–3540 HU) and 1658 ± 90 (1685, 1513–1756 HU) were detected in the 135 kVp conventional CT dataset for the *T. rex* and *Bos taurus*, respectively. Applying the DECT algorithm for the detection of calcium, generated material maps demonstrated more than a twofold higher concentration of calcium in the *T. rex* compared to the *Bos taurus* specimen while both showed a homogenous distribution of calcium throughout bone (3335 ± 404 HU; 3492, 2476–3739 HU vs. 1445 ± 93 HU; 1500, 1309–1538 HU; Figs. 3 and 4). Furthermore, the material map for fluorine demonstrated the absence of fluorine in the leg bone (1 ± 4 HU; 0, 0–14 HU). Meanwhile the *T. rex* specimen demonstrated a less homogenous and only moderate fluorine content within the bone (1809 ± 296 HU; 1794, 1422–2375 HU) as compared to the calcium material map. Thus, DECT imaging detected a higher amount of calcium and fluorine in the *T. rex* than in the *Bos taurus* specimen *(*p < 0.001).

### Fluorine-based detection of permineralized bone in sediment-embedded fossilized test objects

*Morphology of the sediment-embedded test objects*. Cross-sectional CT imaging of the four test objects, including specimens of *Nothosaurus* sp., Ichthyosauria indet., *Dysalotosaurus lettowvorbecki* and Alcelaphinae indet., demonstrated primarily well-discernable bone structures, with each specimen being partially embedded in sediment (Figs. 1 and 3). Each object differed in bone structure, ranging from a homogenous hyperdense bone structure (*Dysalotosaurus lettowvorbecki* and Alcelaphinae) to heterogeneous bone structures with hyper- and hypodense foci (*Nothosaurus* sp. and ichthyosaur). Additionally, the sediment differed in its texture and density among the different objects.

#### Material maps and quantitative DECT measurements

The results of the quantitative DECT measurements are shown in Fig. 4. The calcium maps demonstrated a moderate to high concentration in the bony structures of the four test objects (2464 ± 441 HU; 2518, 1383–3332 HU) and almost undetectable to moderate concentration levels in the attached sediment (1339 ± 635 HU; 1509, 336–2336 HU), resulting in a significant difference in calcium concentration between bone and sediment (p = 0.025).

**Figure 5:**
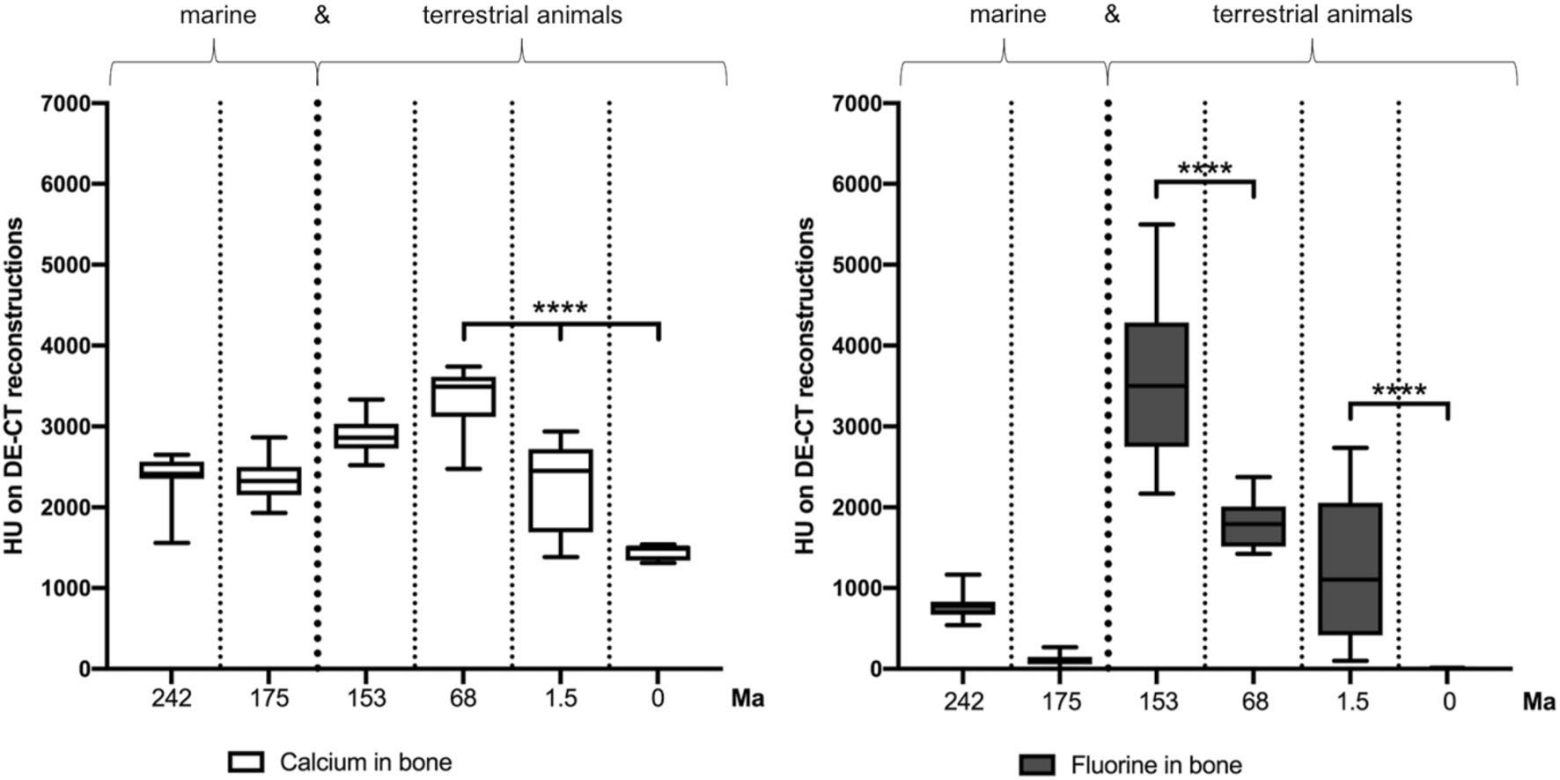
Correlation of geological age with calcium and fluorine contents in bone. In the horizontal, the objects are divided into marine and terrestrial animals and listed in order of their geological time periods. Quantitative assessment of detected calcium and fluorine in DECT reconstructions in bone relative to their age. Significant differences are indicated by * between the fluorine content in bone relative to the specimen age (****p < 0.001).

While generally no fluorine was detected in the sediment (31 ± 168 HU; 0, 0-1059 HU), the bone demonstrated a wide range of fluorine contents (1424 ± 1493 HU; 798, 0-5498 HU), from extremely high values in *Dysalotosaurus lettowvorbecki* to barely detectable in the ichthyosaur. However, the differences in fluorine concentrations between bone and sediment were significant in all specimens (p ≤ 0.003) except for the ichthyosaur specimen (Fig. 4). Interestingly, the fluorine and calcium contents in the marine reptiles *(Nothosaurus* sp. and Ichthoysauria indet.) were generally low, with no significant differences in material contents. Furthermore, the specimens of terrestrial animals (*Bos taurus*, Alcelaphinae indet., *T. rex*, and *Dysalotosaurus lettowvorbecki*) showed an increasing fluorine content with geological age, with significant differences between the geologically older specimens (*Dysalotosaurus lettowvorbecki* and *T. rex*) and the younger specimens (Alcelaphinae and *Bos taurus*), with p values of < 0.001 (Fig. 5). Although the difference between the *T. rex* and Alcelaphinae specimens was not statistically significant (p = 0.17), a substantial decrease in fluorine was noted in the Alcelaphinae specimen. Furthermore, a significant difference between the calcium content of the *T. rex* specimen and the Alcelaphinae and *Bos taurus* specimens was detected (p < 0.001). Notably, while surface artefacts were more pronounced in fluorine material maps, they were independent of the concentration of fluorine and calcium and had no observable effect on the measurements as ROIs were placed specifically in artefact-free areas of the object.

### Advanced pathology characterization of the diseased left dentary using nondestructive DECT-based fluorine-mapping

**Figure 6:**
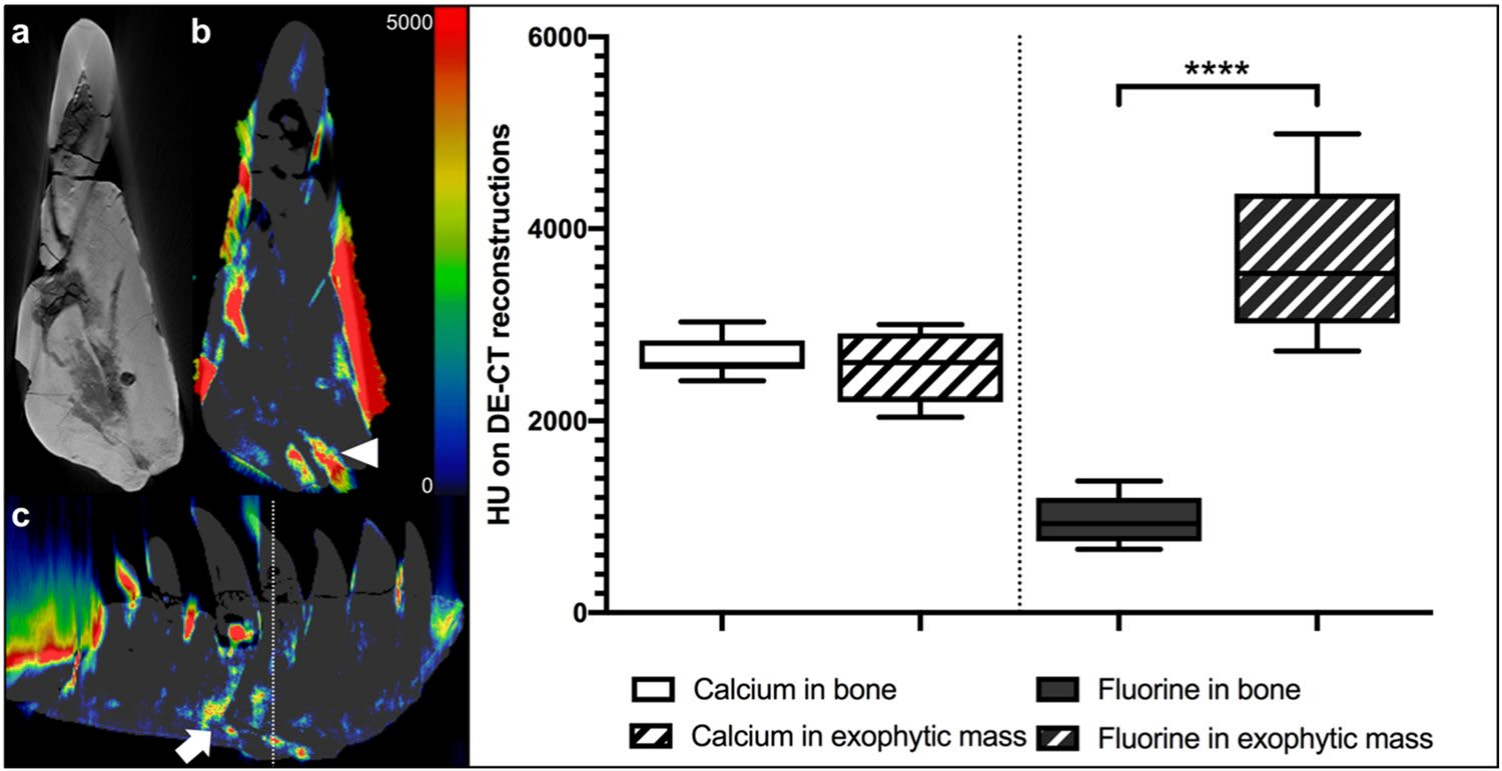
Advanced disease characterization in the left dentary of *Tyrannosaurus rex* using DECT-based fluorine quantification. Conventional CT reconstruction (**a**) and DECT-based fluorine map (**b**) of the left dentary in coronal plane at the level of the focal exophytic mass showing significant fluorine accumulation in areas of reduced bone density (arrowheads). DECT also revealed increased fluorine accumulation at the base of the tooth root and a connecting fluorine deposit in a fistula-like structure in the sagittal plane (**c**; arrow). Red indicates high concentrations of fluorine measured in Hounsfield units (color bar in the upper right corner) whereby the signals on the surface are due to beam hardening artefacts. The Whisker plots show that fluorine content in the exophytic mass was higher than that of the surrounding healthy bone tissue (p < 0.001), while the calcium content did not show any differences. The white dotted line indicates the cross-sectional plane of images (**a**) and (**b**).

The left dentary of the *T. rex* (Figs. 2 and 6) shows two notable findings on visual inspection and CT imaging, a diffuse thickening of nearly the entire left dentary with moderate calcium concentrations (2659 ± 194 HU; 2579, 2412–3024 HU) and a focal exophytic mass on the ventral surface of the dentary ranging from the 3rd to the 5th tooth root with a central accumulation of fluorine (970 ± 242 HU; 927, 660–1374 HU vs. 3700 ± 769; 3533, 2723–4988 HU; p < 0.001). Furthermore, the focal exophytic mass showed diminutive diffuse lucencies extending from the surface to the 5th replacement tooth root and demonstrated a tapering shape with a fistular-like center, which also demonstrated a high fluorine accumulation respectively (p < 0.001). Anatomical structures within the mass are unaffected, as several neurovascular canals pass through the structure. Notably, the remaining areas of bone with increased fluorine accumulation were regions with disturbed bone structure or reduced bone density (Fig. 6). Those imaging features are considered indicative of osteomyelitis, an osseous infection often accompanied by bone swelling and destruction.

## Discussion

This study introduces DECT imaging as a nondestructive technique to analyze elementary compositions and provide morphological and molecular information on highly dense fossilized tissue. Specifically, the newly developed and validated DECT-based calcium and fluorine mapping proved feasible in five extinct and one extant taxa, showing a significantly higher concentration of fluorine in fossilized compared to unfossilized bone and fossilized bone compared to surrounding sediment in terrestrial specimens. Moreover, when applied on a diseased jaw bone of a *T. rex*, fluorine quantification supported the identification and diagnosis of osteomyelitis, revealing the potential of quantitative DECT to characterize osseous diseases in paleopathology.

For the nondestructive analysis, a custom-made DECT three-material decomposition algorithm was applied in extant and fossilized bone specimens, using calcium and iron as calibration materials for the detection of fluorine. Although tailored to the *T. rex* specimen, this algorithm proved feasible and reproducible in all terrestrial fossil test objects. This observation can be explained by the diagenetic stages including the substitution of hydroxyapatite by fluorapatite and permineralization including the deposition of iron in early stages^35^. In addition, fluorapatite and quartz, which were both detected in the fossilized *T. rex* haemal arch, are common permineralization minerals and known features of bone fossilization. Therefore, the calibration materials used here can be assumed to be present in most terrestrial fossil objects.

The calcium and fluorine contents detected by DECT in this study correlated well with the results of the multimodal bone analysis and match existing knowledge of bone taphonomy^36^. Specifically, DECT revealed a significantly higher fluorine concentration in terrestrial fossils as compared to the calcium-containing sediment in which the bones were embedded, thereby revealing fluorine as an imaging indicator for fossilized bone. As opposed to terrestrial fossils, marine animal bones (*Nothosaurus* sp. and Ichthyosauria indet.) showed rather low concentrations of fluorine in the bone and sediment, which may be explained by the diagenetic stages that differ from those of terrestrial animals resulting in only minor deposition of fluorine or iron in the bone.

Notably, the calcium DECT reconstructions also showed significant differences between bone and surrounding sediment in the four test objects. However, similar to the conventional CT images, the visual differentiation of these two structures on calcium material maps can be challenging when there is a significant, but only small difference of the HU values of bone and sediment as demonstrated in the *Dysalotosaurus lettowvorbecki* (Figs. 3 and 4). On the contrary, there is a decisive and visually obvious difference in the fluorine contents of bone and sediment on the DECT-based fluorine maps, suggesting fluorine as a more specific and more apparent indicator of fossilized bone.

Furthermore, fluorine dating is an established method in archaeology due to the incorporation of fluorine during diagenetic processes^7^. Fluorine dating involves measuring the amount of fluoride absorbed by bones to determine their relative age^7,37,38^. Accordingly, the DECT material maps also revealed a significant change in the fluorine concentration with the age of the terrestrial fossil objects (Fig. 5). The fluorine content increased with geological age, being the highest in *Dysalotosaurus lettowvorbecki* (153 Ma) and the lowest in *Bos taurus* (extant), with values of approximately 3500 HU and 0 HU in the DECT reconstructions, respectively. However, one should keep in mind that each fossil sample obviously have undergone a specific diagenetic process as fluorine (including all other elements) is known to be taken up by fossil (as well as extant) bone in different quantities depending on locality, type and condition of bone, as well as the diagenetic setting. Nonetheless, these findings potentially support the development of a novel, nondestructive and fast method for the determination of the relative age of terrestrial fossil objects using DECT imaging in the field of paleontology.

In addition, our study revealed that fluorine mapping has the potential to serve as a diagnostic tool in paleopathological research. Specifically, DECT imaging was applied on the left dentary of the *T. rex*, which shows a tumor-like exophytic mass. This mass was found to demonstrate several distinctive findings on conventional CT imaging, including the preservation of osseous landmarks such as neurovascular canals, lytic areas, and reactive hyperostosis, all of which suggest the diagnosis of an infectious rather than a neoplastic pathology. Based on the hypothesis that fluorine-containing groundwater penetrates the bone during fossilization, the significantly increased fluorine deposition in the central part of the mass suggests an increased permeability and thereby supporting tumefactive osteomyelitis as the most likely diagnosis of this osseous disease. Furthermore, DECT imaging revealed a fistular-like structure connecting the mass to the root of the 5^th^ replacement tooth, which could potentially indicate an odontogenic focus of infection. Finally, peripheral regions of the lesion demonstrated a homogenous distribution of calcium and fluorine, suggesting a periosteal reaction with new bone formation, which is a commonly known mechanism observed during chronic inflammation^39^.

This proof-of-concept study has the limitations of including only a few objects and multimodal bone analysis for only two calibration objects. However, five test objects were included to prove the feasibility and reproducibility of the DECT algorithm. Further chemical analysis and standardization of the test objects would have increased the validity of the developed DECT algorithm, but due to the uniqueness of the objects and museum regulations additional invasive sampling was not possible. In addition, as most specimens were relatively large, the parallel scan of an internal reference phantom was not possible. However, calibration was performed beforehand with carefully selected materials. Therefore, we were not able to prove a correlation between the DECT-measured fluorine content and the actual element content measured by established analyses beyond an apparently reasonable relation. In principle, DECT also allows a three-dimensional analysis of the generated material maps, yet, the DECT algorithm has been developed using a clinical CT scanner and clinical DECT postprocessing software, which is susceptible to artefacts in very large, dense objects and metal. Unfortunately these surface artefacts impeded the reconstruction of pleasant 3D-images. Thus, further investigations should follow addressing the impact of beam-hardening artefacts, which are particularly difficult to account for in complex and highly heterogeneous samples such as paleontological objects. However, as opposed to micro-CT and experimental settings of DECT in geology and mineralogy, the use of clinical CT scanners enables cost-effective, time-efficient, and nondestructive examination of relatively large objects^13,32^ and makes the method generally easily available to researchers worldwide. While this study presents a three-material decomposition algorithm specifically tailored for fluorine, it can be assumed that other elements can potentially be investigated with a suitable and adapted DECT algorithm. However, the results are only valid if the three materials considered in the calculations are the most relevant for X-ray attenuation within the examined volume. Nonetheless, the developed fluorine-specific DECT model should also be applicable to other terrestrial fossil objects, as diagenetic stages include substitution of hydroxyapatite by fluorapatite and permineralization stages including the deposition of iron as mentioned above. In addition, although only small amounts of iron were present in the *T. rex* specimen (0.57 wt. %), it was sufficient for use as a calibration material due to its extremely high HU values (24,422 HU at 80 kVp and 18,311 HU at 135 kVp). While other elements could potentially influence the measurements, another specifically tailored algorithm could account for them, when deemed necessary. Furthermore, although DECT is considered a quantitative imaging method in medicine and allows measurement of the Z_eff_, no further molecular information can be derived from the scan, and its ability to actually quantify is limited compared to other, more invasive techniques such as LA-ICP-MS or electron microprobe imaging. Finally, the hypothesis that quantitative DECT imaging of fluorine can serve as a diagnostic tool in paleopathology should be further investigated using fossil objects of different ages and locations. In particular, fluorine quantification did not prove feasible in marine fossils. In this context, the study of the elements strontium, barium, and cerium may be of interest as an indicator of marine fossilized bone. The uptake of these elements into biological apatite and their applicability as a promising chronometer analogous to fluorine uptake was demonstrated previously in marine organisms such as Mesozoic and “Tertiary” fish, Cretaceous marine reptiles, Devonian conodonts, and Silurian thelodonts^40–42^.

In conclusion, this study highlights the relevance of radiological imaging techniques in the natural sciences by introducing quantitative DECT imaging as a nondestructive approach for density- and element-based material decomposition in paleontological research. Specifically, DECT-based fluorine quantification allowed for the differentiation of bone and sediment and correlated with the geological age of the investigated terrestrial fossils. DECT further showed potential to assist in the characterization of osseous diseases in unique fossil objects while maintaining their integrity.

## Supplementary Information

Supplementary Information.

## Data Availability

The specimens investigated are housed at the Museum für Naturkunde Berlin, Germany under the following collection numbers: MB.R. 5742.1 (*T. rex)*, MB.Ma. 52910 (*Bos taurus),* MB.R. 2830 (*Nothosaurus* sp.), MB.R. 3538.3 (Ichthyosauria indet.), MB.R. 1317 (*D. lettowvorbecki)*, and MB.Ma. 51906 (Alcelaphinae indet.). The *T. rex* skeleton “Tristan Otto” is privately owned by Niels Nielsen and Jens Peter Jensen. However, a small number of bones that were not used in the mounted skeleton or that have come off when assembling including the investigated haemal arch were donated to the Museum für Naturkunde Berlin and are therefore accessible. In addition, access to all CT data sets of the investigated specimens are provided by the Museum für Naturkunde Berlin, Germany and are publicly available via https://www.museumfuernaturkunde.berlin/en/science/infrastructure/collection/online-collections. All CT images of the herein investigated *T. rex* MB.R.91216 are archived in the Museum für Naturkunde and accessible through the Department of Science Data Management under https://doi.org/10.7479/hyek-4pt0 (Asbach et al., 2018; Clinical CT dataset *Tyrannosaurus rex* MB.R.91316. [Dataset], shared under a CreativeCommons CC-BY-NC 4.0 international license). All other specimens investigated and presented in this study are archived in the Museum für Naturkunde Data Repository under https://doi.org/10.7479/bc32-b573 (Hamm et al. 2022; Quantitative Dual-Energy CT as a nondestructive tool to identify biosignatures in fossil diapsid and mammalian bones [Dataset], shared under CreativeCommons CC-BY license).

## Abbreviations

HU: Hounsfield units
DECT: Dual-energy CT
Z_eff_: Effective atomic number
*T. rex*: *Tyrannosaurus rex*
ROI: Region of interest
