# Supplementary material for: Quantitative dual-energy CT as a nondestructive tool to identify indicators for fossilized bone in vertebrate paleontology
Source: Sci Rep. 2022 Sep 30;12:16407. doi: 10.1038/s41598-022-20707-5 (PMC9525674; doi:10.1038/s41598-022-20707-5)
Supplement: Supplementary file 1 — Supplementary Information. [file 41598_2022_20707_MOESM1_ESM.docx]

Title:

Quantitative Dual-Energy CT as a Nondestructive Tool to Identify Indicators for Fossilized Bone in Vertebrate Paleontology

**Authors:**

Charlie A. Hamm, Oliver Hampe, Jürgen Mews, Christina Günter, Ralf Milke, Florian Witzmann, Lynn J. Savic, Lutz Hecht, Sabine Meister, Bernd Hamm, Patrick Asbach, Torsten Diekhoff

**Supplementary Information**

**Materials and Methods of the Multimodal Bone Analysis**

*Bone Defragmentation*

A portion of the haemal arch of *T. rex* and the leg bone of *Bos taurus* were pre-crushed with a mineral splitter and then ground with the centrifugal ball mill S1 (Retsch GmbH). A Sinterkorundmahlbecher was then used for grinding. This process was repeated until the bones were present as fine powder. Bone defragmentation was carried out at the Museum für Naturkunde Berlin, Germany.

*X-ray Diffractometry (XRD)*

X-ray powder diffraction was performed on a Panalytical Empyrean diffractometer by using low-background silicon single-crystal holders housed at the Freie Universität Berlin, Germany. The analysis was performed by Cu-Kα radiation excited at 40 mA/45 kV. The diffraction angle was measured from 5 – 95° at a step size of 0.013° (2Θ) with a counting time of 200 s per step. Graphs and certain peak patterns derived from the XRD analysis provide qualitative information on the crystallized and amorphous composites of the investigated calibration objects. The results were evaluated by High Score Plus.

*Scanning Electron Microscopy (SEM) Coupled with Energy Disperse X-ray Spectrometry (EDX)*

SEM was performed on a JEOL JSM 6510 SEM instrument with a tungsten hairpin filament (15 kV) equipped with an EDX spectrometer (INCAx-act SN detector) produced by Oxford Instruments and housed at the University of Potsdam, Germany. For SEM observations, small amounts of bone fragments were placed on carbon tape and coated with a 25 nm thick layer of carbon with a Polaron CC7650 carbon coater. A back-scattered electron (BSE) detector was used for material contrast images, and a secondary electron (SEE) detector was used for topographic images at 15 kV. The program INCA was used to determine the fluorine content of the samples. The generated microscopic images and read-out spectra derived from the SEM instrument coupled with EDX provided qualitative and semiquantitative information on the crystallized and amorphous composites of the investigated calibration objects.

*Optical Emission Spectrometry (ICP-OES) and Elemental Analysis (EA)*

An Agilent 5100 ICP-OES equipped with a vertical dual view (VDV) was used for the determination of major, minor and trace elements in the *T. rex* specimen and leg bone of *Bos taurus* at the University of Potsdam, Germany. Thirty milligrams of the fine powder was digested in a mixture of 1 ml of concentrated HNO_3_ and 0.5 ml of concentrated HClO_4_ and evaporated almost to dryness. The evaporated solution was supplemented with HNO_3_ up to 15 ml. In contrast to the literature^1^, the sample was not incinerated but dissolved with collagen and measured with ICP-OES.

The sulfur content was determined at the GeoForschungsZentrum Potsdam, Germany, with an Eltra CS 2000. The sample was weighed in ceramic boats and burned in an oxygen stream. Sulfur was measured as SO_3_ with an infrared measuring cell. The elementary analysis determining the concentrations of nitrogen, carbon and hydrogen in the solid samples was carried out using a Euro EA 3000. Powders were packed into tin foils digested by oxidative combustion. Combustion occurred explosively in a highly oxygen-enriched helium atmosphere. The EA was performed at the University Potsdam, Germany.

**Results of the Multimodal Bone Analysis**

*XRD*

XRD analysis of the *T. rex* haemal arch MB.R. 5742.1 revealed a well-crystallized sample with a low and straight background signal, which was identified as fluorapatite (francolite; JCPDF 98-008-4227; **Fig. S3**). A small amount of quartz (SiO_2_, JCPDF 98-0150-6198) was visible with characteristic peaks in the investigated 2Θ region. In addition, the sharp and well-developed diffraction reflexes indicate good crystallinity of the phases. The XRD analysis of the leg bone of *Bos taurus* showed a high and curved background and less well-developed diffraction reflexes than the *T. rex* specimen (**Fig. S4**). The patterns of the leg bone were identified as calcium-deficient hydroxyapatite (CdHA; JCPDF 98-009-3783). The morphology of the background exhibited a high proportion of X-ray amorphous phases, and the shape of the reflexes indicated poorly crystallized, non-stoichiometric nano-particles, characteristic of so-called biogenic apatite crystallites typical in animal hard tissue.

*SEM Coupled with EDX*

The *T. rex* specimen appeared well shaped with hexagonal habits (Fig. S3). It did not show any porosity, and the fragments were larger than those of the leg bone of *Bos taurus*. The surfaces were smooth with sharp edges. The fluorine content of the *T. rex* specimen varied between 3.80 and 4.56 percent by weight (wt.%), with an average of 4.16 wt.%. The leg bone consisted of randomly oriented poorly formed plate-shaped bone fragments. They appeared in thin layers of tightly interlocked fluffy flakes with a high surface area. The fluorine content of the leg bone was below the detection threshold.

*ICP-OES and EA*

The quantitative chemical analyses of both calibration objects revealed substantial differences in composition (Table 1). Referring to the XRD, SEM, and EDX results, the calculation of the crystal phases of the *T. rex* and the *Bos taurus* was based on the assumption that the apatite phases were francolite (Ca_5_(PO_4_, CO_3_)_3_F) and hydroxyapatite, respectively. The phase fraction was determined on the basis of calcium for the *T. rex* specimen and phosphorus fraction for the *Bos taurus* specimen. The *T. rex* haemal arch revealed an apatite phase of 96.40 wt. %, a total fluorine content of 4.16 wt. %, and iron content of 0.57 wt. %. The total amount of apatite phase, fluorine, and iron in the *Bos taurus* leg bone was 75.57 wt. %, <0.05 wt. % and 0.01 wt. %, respectively. In addition, the stoichiometric calcium content of the leg bone was slightly higher than the actual measured calcium content, supporting the assumption that the phosphorus phase represented calcium-deficient hydroxyapatite. Since not all of the measured hydrogen could be incorporated into the hydroxyapatite lattice, it was attributed to the organic content, similar to nitrogen and carbon.

The organic content (C, N, and H) of the leg bone was naturally much higher than that of the *T. rex* specimen due to the relatively high percentage of collagen in non-permineralized bony tissue. Both samples were rich in minor and trace elements. However, except for magnesium, which was four times higher in the *Bos taurus*, the amounts of minor and trace elements were much higher in the *T. rex* specimen (Table 1).

**Supplementary Figures**


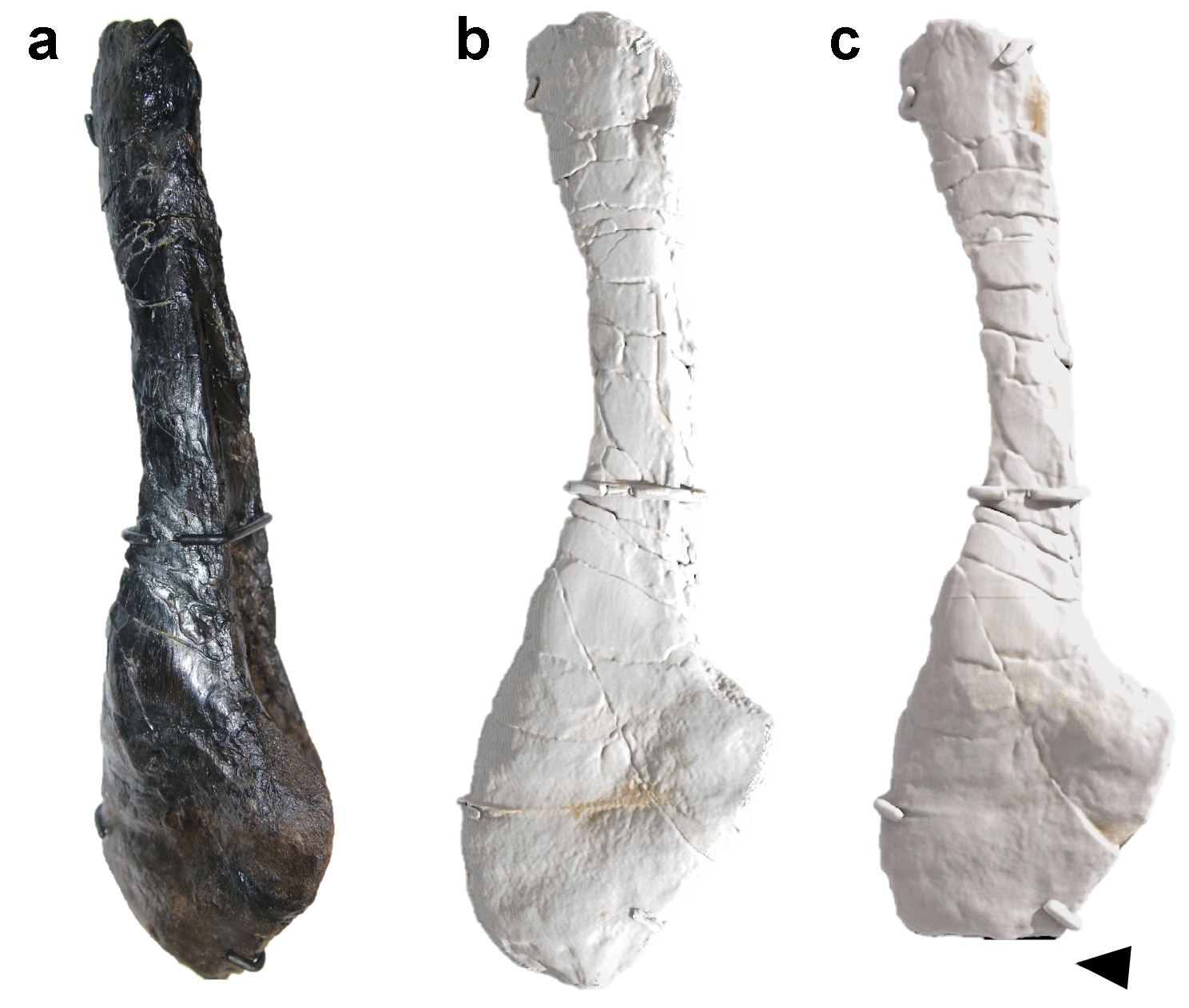


**Figure S1. Photograph and CT-based 3D surface reconstruction of the haemal arch of *Tyrannosaurus rex*, MB.R. 5742.1.** Photograph (**a**) and CT-based 3D surface reconstructions of the haemal arch before (**b**) and after (**c**) the removal of the investigated bone fragment. The place of bone fragment removal is indicated with a black arrowhead.


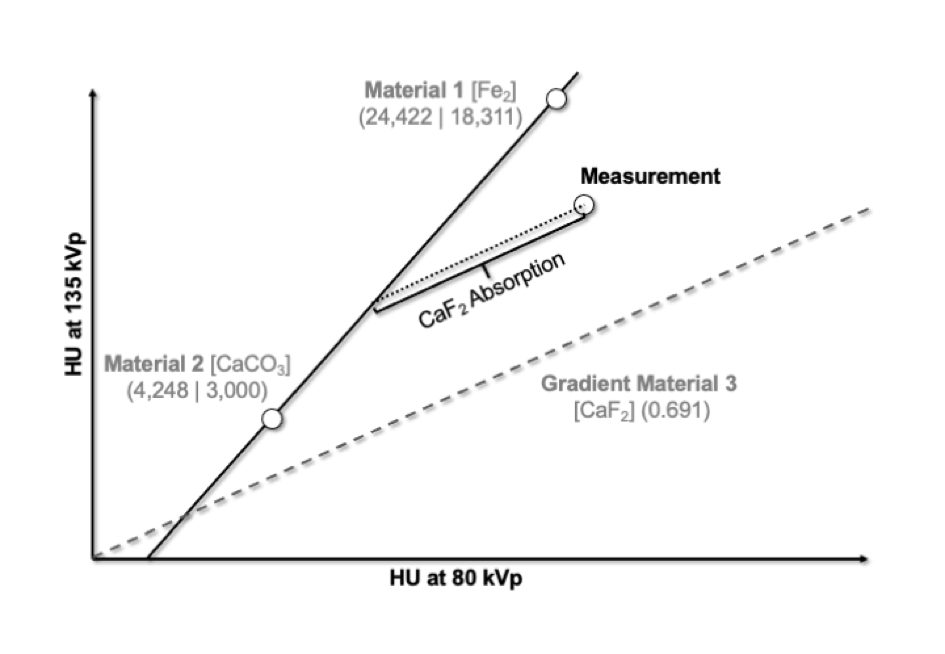


Figure S2. Graphical approach to three-material decomposition in dual-energy CT. Based on the material formulas of two elements, i.e. the absorption in low- and high-energy scans (HU values at 80 kVp | HU values at 135 kVp) and the dual-energy gradient of a third element, the contribution of this third material to the overall absorption is calculated by the distance of each voxel on the dual-energy graph from the connecting line between material 1 and 2 measured parallel to the gradient of material 3. This value is displayed on the respective material map (e.g., the fluorine material map in Fig. 3).

**Figure S3. Comparison of extant and fossilized bone matrix using scanning electron microscopy.** Bone matrix composition of **a**) the *Bos taurus* specimen MB.Ma. 52910 and **b**) the *Tyrannosaurus rex* specimen MB.R. 5742.1, along with high-resolution electron images.

**
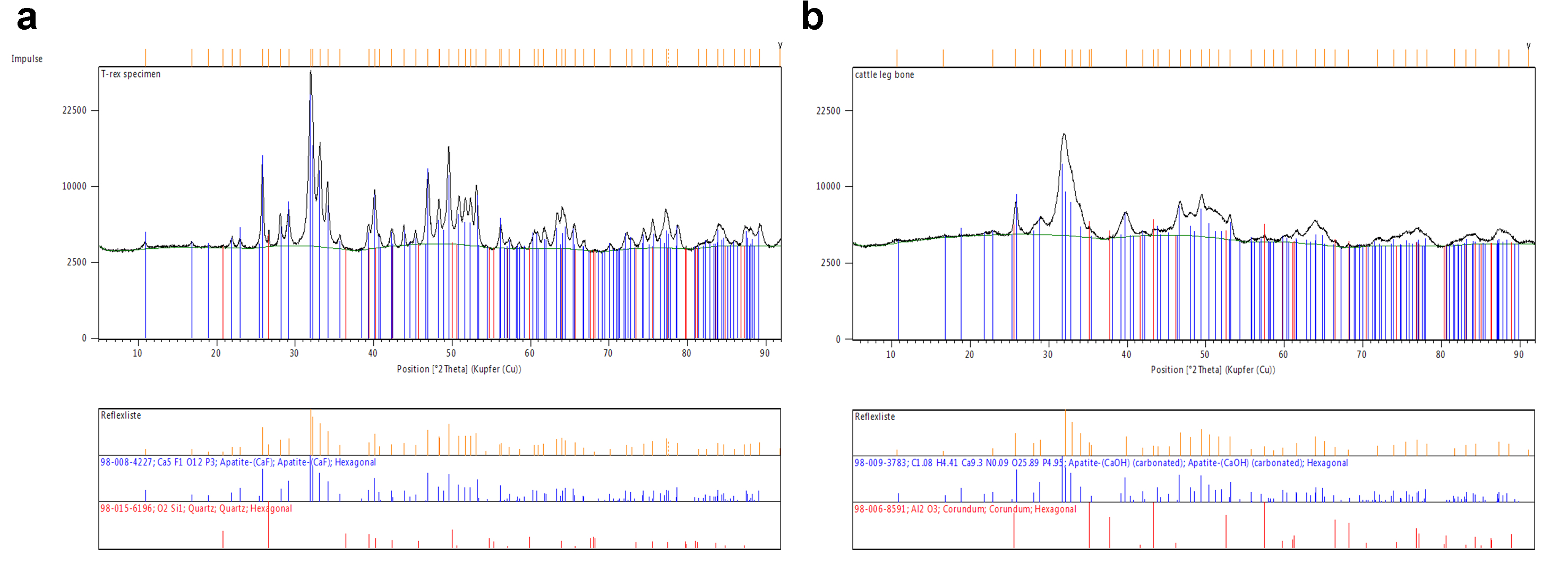
**

**Figure S4. Comparison of fossilized and extant bone matrix using X-ray diffractometry.** The diffraction peak pattern of **a**) the *Tyrannosaurus rex* specimen MB.R. 5742.1 demonstrates a crystallized sample with a low and straight background and a peak pattern matching fluorapatite and quartz, which are common permineralization minerals and a feature of postmortem bone fossilization. **b**) The leg bone of the *Bos taurus* specimen MB.Ma. 52910 demonstrates a rather poorly crystallized bone matrix and a pattern consistent with calcium-deficient hydroxyapatite.

**Supplementary References**

1 Kisser, W. Quantitative Bestimmung von Blei in Blut, Harn und Knochen mittels inverser Voltammetrie. *Arch. Toxicol.* **34**, 237-241 (1975).
